# The rs1862513 promoter variant of resistin gene influences susceptibility to nonalcoholic fatty liver disease

**DOI:** 10.1590/1806-9282.20231537

**Published:** 2024-07-19

**Authors:** Shadi Nouri, Mahsa Navari, Radmehr Shafiee, Touraj Mahmoudi, Gholamreza Rezamand, Asadollah Asadi, Hossein Nobakht, Reza Dabiri, Hamid Farahani, Seidamir Pasha Tabaeian

**Affiliations:** 1 Arak University of Medical Sciences, School of Medicine, Department of Radiology – Arak, Iran.; 2 Shahid Beheshti University of Medical Sciences, Research Institute for Gastroenterology and Liver Diseases, Gastroenterology and Liver Diseases Research Center – Tehran, Iran.; 3 Tehran University, Faculty of Veterinary Medicine, Department of Clinical Pathology – Tehran, Iran.; 4 Iran University of Medical Sciences, School of Medicine, Department of Internal Medicine – Tehran, Iran.; 5 Iran University of Medical Sciences, Colorectal Research Center – Tehran, Iran.; 6 University of Mohaghegh Ardabili, Faculty of Science, Department of Biology – Ardabil, Iran.; 7 Semnan University of Medical Sciences, Department of Internal Medicine – Semnan, Iran.; 8 Qom University of Medical Sciences, School of Medicine, Department of Physiology and Pharmacology – Qom, Iran.

**Keywords:** NAFLD, Polymorphism, Resistin, RETN

## Abstract

**OBJECTIVES:**

Nonalcoholic fatty liver disease is the term used for a range of conditions in which fat builds up in the liver and exceeds 5% of hepatocytes without inordinate alcohol intake or other causes of lipid accumulation. Regarding the fact that insulin resistance and obesity play key roles in the pathogenesis of nonalcoholic fatty liver disease, as well as the connection between resistin and these metabolic diseases, the association between nonalcoholic fatty liver disease and a resistin gene (*RETN*) polymorphism was examined.

**METHODS:**

In this genetic case–control association study, 150 biopsy-proven nonalcoholic fatty liver disease patients and 154 controls were enrolled and genotyped for the *RETN* rs1862513 (-420C>G) gene polymorphism using PCR–RFLP method.

**RESULTS:**

The −420C>G genotype frequency distributions in both groups were consistent with Hardy-Weinberg equilibrium (HWE; p>0.05). The carriers of the *RETN* −420C>G "CC" genotype compared with the "GG" genotype occurred less frequently in the cases with nonalcoholic fatty liver disease than in the controls, and the difference remained significant even after adjustment for confounding factors (p=0.030; OR=0.47, 95%CI=0.36–0.93). Interestingly, the *RETN* −420C>G "C" allele was also associated with a decreased risk for nonalcoholic fatty liver disease too (p=0.042; OR=0.72, 95%CI=0.53–0.95).

**CONCLUSION:**

We found for the first time an association between biopsy-proven nonalcoholic fatty liver disease and *RETN* −420C>G promoter polymorphism. The carriers of the *RETN* −420C>G "CC" genotype had a 53% decreased risk for nonalcoholic fatty liver disease. Our findings, however, need to be corroborated by further studies.

## INTRODUCTION

Nonalcoholic fatty liver disease (NAFLD) is the term used for a range of conditions in which fat builds up in the liver and exceeds 5% of hepatocytes without inordinate alcohol intake or other causes of lipid accumulation. NAFLD affects roughly 25% of adults worldwide and encompasses simple steatosis, nonalcoholic steatohepatitis (NASH), and cirrhosis^1^. Positive associations between NAFLD and circulating insulin levels, insulin resistance (IR), type 2 diabetes (T2D), and obesity have been found. Moreover, patients with NASH have a higher IR index than cases with simple steatosis, and the severity of the increased levels of liver enzymes is also higher in NAFLD patients with IR than those without IR. Finally, significant associations between variants in insulin signaling pathway genes including insulin receptor (*INSR*), insulin receptor substrate 2 (*IRS2*), insulin-like growth factor 1 (*IGF1*), and insulin-like growth factor binding protein 3 (*IGFBP3*) and the risk of NAFLD have been discovered^2-7^.

Resistin, a 12-kDa cysteine-rich polypeptide hormone protein, is the product of the *RETN* gene and may be involved in the pathogenesis of NAFLD. It is predominantly secreted by macrophages and adipocytes and has a pivotal role in energy homeostasis. Resistin inhibits the ability of insulin to stimulate cellular glucose uptake and links obesity to insulin resistance^8^. IR, obesity, and NAFLD are all associated with alterations in circulating resistin levels. It has been demonstrated that resistin levels were positively associated with body mass index (BMI)^9^, IR^10^, and NAFLD^11^. Previous reports have also indicated significant associations between *RETN* gene polymorphisms and the expression of *RETN* gene^12^, serum resistin levels^13^, and obesity^14^. Thus, this study was designed to investigate the possible contribution of the *RETN* rs1862513 gene polymorphism to NAFLD. The inclusion criteria for selecting this single-nucleotide polymorphism (SNP) include (I) its common use in prior genetic studies; (II) functional importance; and (III) relatively high degree of heterozygosity.

## METHODS

### Study population

After informed consent, 150 patients with biopsy-proven NAFLD (age range, 32–86 years) and 154 controls (age range, 31–82 years) were enrolled. NAFLD patients were enrolled after fatty liver diagnosis, which in turn was defined by (a) ultrasonographic confirmation of fatty liver, (b) having high circulating levels of AST, ALT, and GGT, (c) excluding subjects with other causes of liver disease such as Wilson's disease, alpha-1 antitrypsin deficiency, viral hepatitis, and alcohol use of more than 70 g/week in women or more than 140 g/week in men, and (d) liver biopsy evidence of NAFLD using the Brunt's criteria by an experienced pathologist. The control group was recruited from the research staff of the Institute and medical students. Those who were free of elevated AST, ALT, GGT, viral hepatitis infection (examined by blood test), had no liver steatosis (examined by abdominal ultrasonography), and were not alcoholic or on regular medications were enrolled as controls. This study complied with the principles of the Declaration of Helsinki and was performed according to the Institute's Ethics Committee approval.

### Genotyping

Genomic DNA purification from 5 mL EDTA-anti-coagulated whole blood was performed using phenol–chloroform extraction and ethanol precipitation. Then, the DNA samples were stored at −20°C. To detect the genotypes of the *RETN* rs1862513 or −420C>G variant, we used PCR–RFLP method. In brief, genomic DNA was amplified using the primers: 5’-TCCTGGCTTGGCTAATAAGTC-3’ and 5’-TACCAGTTCTATTGCTCATGGG-3’ to discover the genotypes of the *RETN* gene. PCR conditions were as follows: (I) pre-degeneration at 95°C for 10 min, (II) 35 cycles for degeneration at 95°C for 45 s, annealing at 61°C for 40 s, and extension at 72°C for 40 s, and (III) final extension at 72°C for 10 min. The PCR products (500 bp) were then analyzed by RFLP: overnight digestion with the restriction enzyme of EarI (Fermentas, Leon-Rot, Germany) at 37°C in a water bath. Electrophoresis was performed on a 2.5% agarose gel stained with ethidium bromide and then the RFLP products (500 bp, 363 bp, and 137 bp) were visualized using ultraviolet light transillumination^15^. The "C" allele of the *RETN* −420C>G SNP had bands of 363 bp and 137 bp, whereas its "G" allele had a band of 500 bp, thus an individual with band(s) at 363 bp and 137 bp, at 500 bp only, or at 500 bp, 363 bp, and 137 bp was defined as "CC" homozygotic genotype, "GG" homozygotic genotype, and "CG" heterozygotic genotype, respectively. To verify the genotyping results, 20% of all the subjects were genotyped twice by different laboratory personnel; the reproducibility was 100%.

### Statistical analyses

T-test was used to compare continuous variables. To compare categorical clinical variables and allele frequencies between the case and control groups, we used chi-square (χ^2^) test. χ^2^ test was also used to examine HWE. To assess the association between the genotype frequencies of the *RETN* −420C>G and the risk of NAFLD, logistic regression analysis was used. This analysis was also applied to adjust the confounding factors such as age and BMI. To evaluate the measure of the associations, the odds ratio (OR) and the corresponding 95% confidence interval (95%CI) were used. A p-value less than 0.05 was considered statistically significant (SPSS, version 25.0, Chicago, IL, USA).

## RESULTS

Table 1 shows the clinical and biochemical data of controls and NAFLD patients. The patients were more likely to be male (p<0.001) and smokers (p=0.02). They also had higher age (p<0.001), BMI (p<0.001), systolic blood pressure (p<0.001), diastolic blood pressure (p<0.001), AST (p<0.001), ALT (p<0.001), and GGT (p<0.001) than the controls.

**Table 1:** Selected characteristics of the patients with nonalcoholic fatty liver disease and controls^a^.

Variables		Patients (n=150)	Controls (n=154)	p-value
Age (years)		38.1 (9.3)	29.0 (7.4)	<0.001
Body mass index (kg/m^2^)		29.4 (5.0)	23.2 (3.3)	<0.001
Sex				
	Men	109 (72.7)	80 (51.9)	
	Women	41 (27.3)	74 (48.1)	<0.001
Smoking history				
	No	111 (74.0)	140 (90.9)	
	Former	19 (12.7)	9 (5.8)	
	Current	20 (13.3)	5 (3.3)	0.020
	Systolic blood pressure (mmHg)	123.8 (15.3)	113.9 (13.4)	<0.001
	Diastolic blood pressure (mmHg)	74.5 (9.8)	70.0 (8.1)	<0.001
	Aspartate aminotransferase (IU/L)	39.4 (17.1)	19.8 (7.1)	<0.001
	Alanine aminotransferase (IU/L)	71.1 (39.9)	19.5 (10.2)	<0.001
	Gamma glutamyl transferase (IU/L)	57.8 (31.4)	19.1 (8.3)	<0.001
Steatosis				
	Grade 0			
	Grade 1	38 (25.3)		
	Grade 2	81 (54.0)		
	Grade 3	31 (20.7)		
Necroinflammation				
	Grade 0	46 (30.7)		
	Grade 1	57 (38.0)		
	Grade 2	45 (30.0)		
	Grade 3	2 (1.3)		
Fibrosis				
	Stage 0	88 (58.7)		
	Stage 1	55 (36.7)		
	Stage 2	7 (4.6)		
	Stage 3			
	Stage 4			

a Variables presented as mean (SD) or number (%).

Both groups had a consistent genotype frequency distribution and presented HWE, hence we used a representative sample population. The carriers of the "CC" genotype of *RETN* −420C>G variant compared with the carriers of the "GG" genotype were associated with a decreased risk for NAFLD, and the difference remained significant after adjustment for confounding factors including age, BMI, sex, smoking status, SBP, and DBP (p=0.030; OR=0.47, 95%CI=0.36–0.93) (Table 2). Furthermore, the *RETN* −420C>G "C" allele in comparison to "G" allele was significantly underrepresented in the cases with NAFLD compared to controls (p=0.042; OR=0.72, 95%CI=0.53–0.95).

**Table 2:** Distribution of resistin gene (*RETN*) rs1862513 polymorphism in the patients with nonalcoholic fatty liver and in the controls^a^.

RETN (rs1862513)		Controls (n=154)	Patients (n=150)	Crude OR (95%CI) p-value	Adjusted OR (95%CI) p-value^b^
Genotype-wise comparison					
	GG	73 (47.4)	86 (57.3)	1.0 (reference)	1.0 (reference)
	GC	43 (27.9)	37 (24.7)	0.87 (0.71–1.58)0.750	0.92 (0.67–1.60)0.890
	CC	38 (24.7)	27 (18.0)	0.43 (0.41–0.88)0.028	0.47 (0.36–0.93)0.030
	GC and CC	81 (52.6)	64 (42.7)	0.72 (0.64–1.20)0.207	0.77 (0.6–1.21)0.219
	CC versus others	38 (24.7)	27 (18.0)	0.61 (0.59–1.23)0.052	0.64 (0.5–1.27)0.056
Allele-wise comparison					
	G	189 (61.4)	209 (69.7)	1.0 (reference)	–
	C	119 (38.6)	91 (30.3)	0.72 (0.53–0.95)0.042	–

a Variables presented as numbers (%).

b Adjusted for age, body mass index, sex, smoking status, systolic blood pressure, and diastolic blood pressure in genotype-wise comparisons.

## DISCUSSION

As a complex disease, NAFLD is caused by the interactions between many environmental and genetic factors, each of which has a somewhat small individual effect; hence, it is difficult to discover them. However, studying the candidate gene polymorphisms can be a useful approach to recognize the potential genes involved in NAFLD pathogenesis, although contradictory findings are not infrequent in genetic association studies. The discrepancies may be explicated by variations in the environmental factors, differences in the disease definition, racial differences in genetic makeup, and statistical analyses^16,17^. Regarding the fact that IR, obesity, and inflammation are of critical importance in the development and progression of NAFLD, and resistin plays an important role in these metabolic disorders, it is biologically reasonable to hypothesize that *RETN* gene may be involved in NAFLD pathogenesis. The release of free fatty acids from adipose tissue and their influx into liver can be accelerated by IR. To maintain glucose homeostasis in patients with NAFLD, insulin secretion seems to increase to make up for low insulin sensitivity in these patients^2-4,18^.

*RETN* gene consists of four exons and is situated on the short arm of chromosome 19. In this study, we demonstrated that there is a significant association between NAFLD and the −420C>G variant located in the promoter of *RETN* gene. The "CC" genotype of *RETN* −420C>G gene polymorphism in comparison to "GG" genotype was a protective factor for NAFLD. The "C" allele of the *RETN* −420C>G variant occurred less frequently in the NAFLD patients too. Alterations in promoter sequence may affect the expression of *RETN* gene and/or the function of RETN protein. The −420C>G promoter variant influences the expression of *RETN* gene. It has been shown that this polymorphism induces resistin mRNA synthesis through the generation of a Sp1/Sp3 binding site^12^, which in turn increases the resistin transcription and finally leads to a higher circulating resistin level. Interestingly, previous studies confirm this hypothesis. The *RETN* −420C>G polymorphism is associated with the expression of *RETN* gene^12^ and serum resistin levels^13,19^. There is a significant association between −420G allele and higher serum resistin levels^13,19^ and obesity^14^. The *RETN* −420C>G "GG" genotype also increases the *RETN* promoter activity^12^. Resistin desensitizes fat, muscle, and liver cells to insulin and causes hepatic insulin resistance^20^. Consistently, resistin levels were positively associated with IR^10^, obesity^9^, NAFLD^11^, and fibrosis severity^21^. Alternatively, resistin may cause NAFLD via inducing inflammation, which is a key contributor to NAFLD pathogenesis. It appears that the link between resistin and inflammatory markers is independent of BMI. Serum resistin levels were positively associated with C-reactive protein (CRP) as an inflammatory biomarker. Resistin is also implicated in inflammatory processes as a proinflammatory factor in liver fibrogenesis and upregulates the expression of proinflammatory cytokines such as TNF-α, IL6, IL12, and MCP-1 in monocytes, macrophages, and hepatic cells through the NF-κB pathway. Resistin also enhances the expression of TNF-α and IL-1β via MEK and ERK signaling pathways by inhibiting some microRNAs^8,22,23^, Consequently, a growing body of evidence corroborates the hypothesis that resistin and its gene, *RETN*, may play a role in the development and progression of NAFLD, and the finding of the present study that the −420C>G polymorphism of *RETN* gene is associated with NAFLD is in line with the above evidence. Notwithstanding these findings, however, the exact molecular mechanism through which the −420C>G variant affects the function of *RETN* gene is largely undetermined and needs to be fully elucidated.

Potential limitations exist in our study. First, it was unreasonable to carry out sub-analyses owing to the modest sample size. Second, due to budget limitations we were unable to measure the circulating resistin level and genotype of more than one variant of the *RETN* gene. Despite the above limitations, the present study had a good design, and we used liver biopsy as the gold standard method to confirm NAFLD diagnosis. Moreover, it also had novel and interesting findings that were consistent with prior research.

## CONCLUSION

Our findings indicated that *RETN*-420C>G "CC" genotype had a 53% decreased risk for NAFLD compared with its "GG" genotype counterpart. Interestingly, this observation is pertinent from a theoretical viewpoint, although further studies with larger samples of different populations are required to elucidate the participation of *RETN* gene polymorphisms in NAFLD susceptibility.
